# Solar power forecasting beneath diverse weather conditions using GD and LM-artificial neural networks

**DOI:** 10.1038/s41598-023-35457-1

**Published:** 2023-05-25

**Authors:** Neetan Sharma, Vinod Puri, Shubham Mahajan, Laith Abualigah, Raed Abu Zitar, Amir H. Gandomi

**Affiliations:** 1 Electrical Engineering Department, BGSB University, Rajouri, India; 2 Ajeenkya DY Patil University, Pune, Maharashtra 412105 India; 3 University Center for Research & Development (UCRD), Chandigarh University, Mohali, India; 4 MEU Research Unit, Middle East University, Amman, Jordan; 5 Hourani Center for Applied Scientific Research, Al-Ahliyya Amman University, Amman, 19328 Jordan; 6 Applied science research center, Applied science private university, Amman, 11931 Jordan; 7 School of Computer Sciences, Universiti Sains Malaysia, 11800 Pulau Pinang, Malaysia; 8 School of Engineering and Technology, Sunway University Malaysia, Petaling Jaya 27500, Malaysia; 9 Computer Science Department, Prince Hussein Bin Abdullah Faculty for Information Technology, Al al-Bayt University, Mafraq 25113, Jordan; 10 Sorbonne Center of Artificial Intelligence, Sorbonne University-Abu Dhabi, Abu Dhabi, United Arab Emirates; 11 Faculty of Engineering and Information Technology, University of Technology Sydney, Ultimo, NSW 2007 Australia; 12 University Research and Innovation Center (EKIK), Óbuda University, 1034 Budapest, Hungary

**Keywords:** Energy science and technology, Engineering, Mathematics and computing

## Abstract

Large-scale solar energy production is still a great deal of obstruction due to the unpredictability of solar power. The intermittent, chaotic, and random quality of solar energy supply has to be dealt with by some comprehensive solar forecasting technologies. Despite forecasting for the long-term, it becomes much more essential to predict short-term forecasts in minutes or even seconds prior. Because key factors such as sudden movement of the clouds, instantaneous deviation of temperature in ambiance, the increased proportion of relative humidity and uncertainty in the wind velocities, haziness, and rains cause the undesired up and down ramping rates, thereby affecting the solar power generation to a greater extent. This paper aims to acknowledge the extended stellar forecasting algorithm using artificial neural network common sensical aspect. Three layered systems have been suggested, consisting of an input layer, hidden layer, and output layer feed-forward in conjunction with back propagation. A prior 5-min te output forecast fed to the input layer to reduce the error has been introduced to have a more precise forecast. Weather remains the most vital input for the ANN type of modeling. The forecasting errors might enhance considerably, thereby affecting the solar power supply relatively due to the variations in the solar irradiations and temperature on any forecasting day. Prior approximation of stellar radiations exhibits a small amount of qualm depending upon climatic conditions such as temperature, shading conditions, soiling effects, relative humidity, etc. All these environmental factors incorporate uncertainty regarding the prediction of the output parameter. In such a case, the approximation of PV output could be much more suitable than direct solar radiation. This paper uses Gradient Descent (GD) and Levenberg Maquarndt Artificial Neural Network (LM-ANN) techniques to apply to data obtained and recorded milliseconds from a 100 W solar panel. The essential purpose of this paper is to establish a time perspective with the greatest deal for the output forecast of small solar power utilities. It has been observed that 5 ms to 12 h time perspective gives the best short- to medium-term prediction for April. A case study has been done in the Peer Panjal region. The data collected for four months with various parameters have been applied randomly as input data using GD and LM type of artificial neural network compared to actual solar energy data. The proposed ANN based algorithm has been used for unswerving petite term forecasting. The model output has been presented in root mean square error and mean absolute percentage error. The results exhibit a improved concurrence between the forecasted and real models. The forecasting of solar energy and load variations assists in fulfilling the cost-effective aspects.

## Introduction

Beneath universal typical weather transformation, exploring renewable energy resources has become the need of the hour. Nevertheless, the main problems allied with non-conventional resources are discontinuous in the environment. Solar irradiance varies following the surrounding topography, altitude conditions^1^, etc. The cooling and heating chiefly depends upon the sun's positioning and locality in the north and south hemispheres. While considering various advantages and shortcomings, power generation forecasting could be a considerable aspect of the prior scheduling of its allocation and production. In area of Artificial Intelligence, Artificial Neural Network system is one of the vital tools for optimization and forecasting etc. which not only saves time but also computes the outcome with more precision in comparison to other conventional techniques (Haykin 1994). This approach is used in the Science and Engineering fields. The energy and mechanical engineering fields, artificial neural network techniques have bagged more eminence recently, which is also embedded with some limitations as well. The solar PV^2^ is impending towards the peak of network uniformity with the consistent diminution in terms of asking price because of the increased PV installations and enhanced capacities in the entire generation system around the globe. Reduced solar PV installation costs have made it of utmost concern to design or prepare some reliable approach^3^ that can help predict things much earlier.

Solar energy harnessing faces certain key hindrances^4^ by weather conditions like cloud coverings, movement of the winds, increased temperature, humidity proportion, etc. Rather than forecasting for more extended periods, it is more effective and fruitful to forecast for a few minutes ranging from 5 to 10 min, before changing factors such as cloud movements, accumulation, and dissipation which are pretty responsible for shading of PV panels ensuing in the impeding of production and supply rates as well^5^. The varied scales used for prediction based on time, such as hourly, daily, and prior are enviable for the activities like grid functioning ramping actions, imminent markets related to power generation, and distributions. The key solar power prediction tools like Global Horizontal Irradiations^6^ on time-based forecasting may be a handy thing to answer this issue. In general, the recent research assessment reveals that the prediction ranging from intermediate to massive levels embraces Numerical Weather Prediction; forecasts dependent on satellite prediction can, be doled out in a more advanced way to modify daily based solar prediction. Forecasting the cloud's progress can be just made using its images. More detailed results can also be obtained using prediction mechanisms^7^ based on the statistical technique for forecasting and observing the weathering conditions. However, it stays scarce due to space and time while achieving conditional eloquence^8^ as solar photovoltaic panels generated output largely depends on irradiance and temperature^9^ variations. A mammoth amount of work to broaden and devise automatic, dexterous, and precise approaches like sizeable modeling, statistical approach, artificial intelligence-based approach, and the amalgam of various techniques to set up the precision of the renewable resources for minuscule solar foretelling is accomplished. The Arithmetic Investigation Methodology combines the input parameters and the parameters to be forecasted, also called LMA. The artificial neural network^10^ can support petite term prediction with plentiful input parameters like wind speed, temperature, tension, and relative humidity proportions. An additional existing substitute is MWNN for forecasting solar irradiance. In order to ascertain the clarification functionality, indexing a new model referred to as the ARF method is also excellent for dumpy forecasting. There are ways to use the data gathered over days, months, and years. The shifting variables and infinitely nonlinear parameters exposed to the environment can be learned. This work's primary goal is to forecast photovoltaic cell output and reduce forecasting errors using an artificial neural network system. The ANNs^11^ are primarily implemented on a broad range of realistic and handy utilization, from process observations, monitoring, defect identification and adaptive individual intrusions to innate proceedings and AI regarding environmental processes and computers^12^.

Principally, the conception of ANNs can be separated into two parts: structural design and efficient characteristics known as neurodynamics. The primary part implies scheming the synthesized neurons' arrangement and composition and pertinent connections. In contrast, the next part encompasses the character of learning, associated with recalling and unremittingly equalization of the new information with previously existing information. The numerous brain mechanisms include the interaction of neurons^13^, also known as inputs, synaptic strength, activation biasing, and output basics. There may be a neuron made artificially to have '*n*' no. of inputs like *X*_*i*_, where *i* = 1, 2, 3, 4, 5…, *n* exhibits input signal source. The source can be neighborhood neurons or environmental structures^14^. All the individual inputs are weighted to accomplish main processing body by weight factor (w_j_), also called connecting force. As a result, the corresponding force transfer signal is analogous to the *w*_*j*_*x*_*j*_ symbolized fragment of a fresh and realistic signal. In contrast, in order to create signal, input signal of that neuron could be past considerable value, say *h*, entire signal allied^15^ with neuron is derived using relationship given below^16^:

1 $$\sum_{i=1}^{n}{x}_{i}{w}_{i}={w}_{1}{x}_{1}+{w}_{2}{x}_{2}+\dots +{w}_{n}{x}_{n}=>total={w}^{T}X.$$

A solar cell's voltage and current characteristics under typical weather no current is obtained when there is no load, and the peak voltage detected across a solar cell is referred to as the open circuit voltage (Voc). In contrast, when a solar cell is short-circuited, the voltage across the cell is at its lowest (zero) value, but the current leaving the cell reaches its highest or peak value^17^. The solar photovoltaic cell operates flawlessly when it reaches MPP, which is the point in the curve where the cell produces the most electrical power. The projected values of Vmp and Imp are Voc and Isc, and a PV cell's output current and voltage are significantly dependent on temperature. However, the wattage varies depending on the surrounding temperature. The relationship shown below can be used to determine the^11^ photocurrent I_ph_.

2 $${I}_{ph}\left(G, T\right)=\left[{I}_{scn}+{K}_{i}(T-{T}_{n})\right]\frac{G}{{G}_{n}},$$

where, I_sc_ is short circuit current, Ki is Coefficient of temperature, Gn represents solar irradiance value = 1000 W/m^2^, and T_n_ is solar cell temperature.

## Arithmetical modelling of ANN for solar output power forecasting

The quandary under concern is the forecasting of solar output power, which is also the desired outcome. The impact on the weighted signal and its correlation with activation function (*f*), the linear or non-linear output^15^ is:

3 $${\text{Output}} = {\text{f }}\left( {{\text{total}}} \right).$$

The input parameters’ values are *t*, *h*, and I_r_ and V is target variable, the learning rate is 0.1, and tan*h* is believed to be activation procedure for artificial neural structure. It includes two hidden layers with 14 neurons, like hi^1^ and hi^2^. The weight akin to input h_i_^1^ is denoted by w_ij_. Similarly, the weights from h_i_^1^ to h_i_^2^ are denoted by θ_ij._ The weights from h_i_^2^ to the output parameters are given by φ_ij_. The mathematical interpretation^18^ is as follows:

4 $${h}_{i}^{1}=\sum_{i=1}^{3}\left(\sum_{j=1}^{14}W\times {X}_{ij}+{b}^{1}\right).$$

X_ij_ denotes the real input values to the hidden layer, W_ij_ is the starting weights assigned as 1, and b1 is biased. The hyperbolic tangent consequent to h_i_^1^ is represented^17^ as follows.

5 $${Oh}_{i}^{1}=\mathrm{tanh}\left({h}_{i}^{1}\right).$$

Here, i = 1, 2, 3…14, and Oh_i_^1^ are the total nodes' actual output. The representation of weighted function θ_ij_, with 14 neurons in the next layer^19^, is given by:

6 $${h}_{i}^{2}=\sum_{i=1}^{14}\left(\sum_{i=}^{14}{\theta }_{ij}\times {Oh}_{i}^{1}+{b}^{2}\right),$$

where, Oh_i_^1^ is consequent output to first hidden layer, θij is initialized weights as one, and b2 is the bias as 1. The relationship can obtain the actual output^12^ of hi^2^:

7 $${Oh}_{i}^{2}=\mathrm{tan}\left({h}_{i}^{2}\right).$$

Targeted output solar power (OV) in the output layer^20^ is given by:

8 $$V=\left(\sum_{i=1}^{14}{Oh}_{i}^{2}\times {\varphi }_{i}\right)+{b}^{3}.$$

Tangent function for the targeted value^15^ is represented by:

9 $$OV=\mathrm{tanh}\left(V\right).$$

The relationship can complete error^21,22^ computations:

10 $$E=\frac{1}{2}\sum_{j=1}^{n}{\left({VE}_{j}-{OV}_{j}\right)}^{2},$$

where, the value of 'n' denotes the output of the neurons and VE represents actual output solar power. The up gradation of weights is performed using the relationships^23–25^ as follows:

11 $${w}_{ij}^{2}={w}_{ij}^{1}+{\Delta }_{ij},$$

12 $${\theta }_{ij}^{2}={\theta }_{ij}^{1}+{\Delta \theta }_{ij},$$

13 $${\varphi }_{i}^{2}={\varphi }_{i}^{1}+{\varphi }_{i}^{1}+{\Delta \varphi }_{i}.$$

Here, ΔW_ij_, Δ_θij,_ and Δφ_i_ can be explained as under^26–28^:

14 $${\Delta W}_{ij}=-\eta \frac{\delta E}{{\delta W}_{ij}},$$

15 $${\Delta \theta }_{ij}=-\eta \frac{\delta E}{{\delta \theta }_{ij}},$$

16 $${\Delta \varphi }_{i}=-\eta \frac{\delta E}{{\delta \varphi }_{i}}.$$

Upgrading weights in its whole continues until the fault is minimised. In a swarm of neural networks, every single lone neuron is referred to as a node, and each output simply behaves like its succeeding neuron's input. In general, the exploitation above might function for each node in a network with "n" number of nodes. Each neuron can be distinguished from the others by the subscript I depending on output and non-linearity, if function, active signals, weights, and inputs are represented^17^ by the subscript "i". A sigmoid and stiff limiting facet may also be a part of the very significant non-linear feature. The values fall between 0 and 1. Since the sigmoid function is thought to have non-linearity, monotonicity, an uncomplicated origin, and bounded-like properties, it has gained greater attention for the cause^29^. By the process of learning, the weights and neurons contain useful information. The BPA is said to be the most sophisticated method among the many proposed methods for getting closer to the weights between the input and output. The connection allows for updating each weight during iterations^13,14^:

17 $${w}_{ij}\left(N\right)={w}_{ij}\left(P\right)-\eta \frac{\Delta E}{\Delta {w}_{ij}}.$$

Here, w_ij_ symbolizes the ith and jth neuron, and ƞ (0 and 1) and E indicates the error and can be calculated by the use of the following relationship^14^:

18 $$E=\sum_{k=1}^{l}\sum_{j=1}^{q}{({b}_{kj}-{z}_{kj})}^{2},$$

where b_kj_ z_kj_ are real and expected values for the neural network. This resemblance applies approximately four signals in conjunction with two invisible layers projecting fifth signal using neural model.

## Artificial neural network system

**Figure 1:**
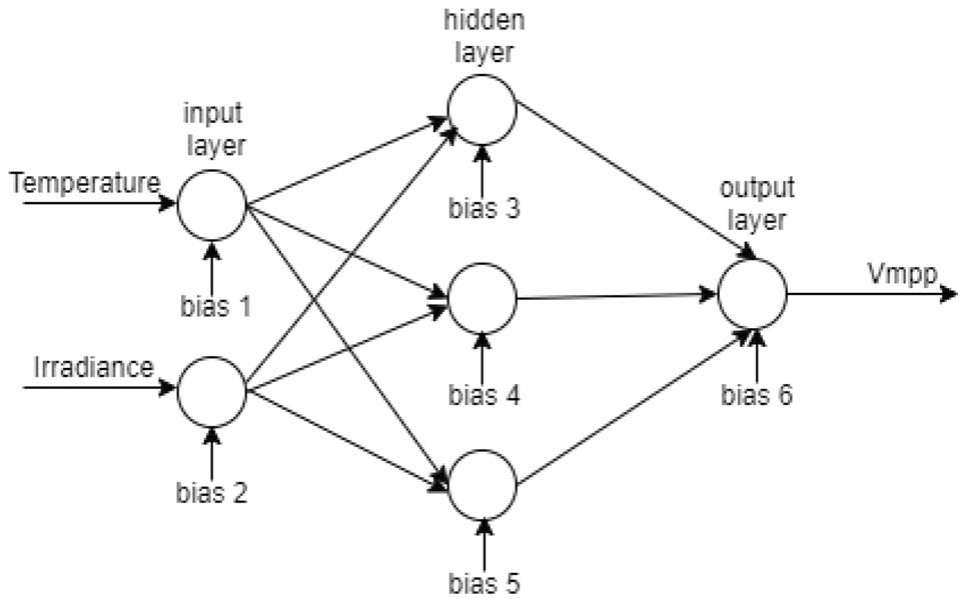
Structure of a neural network system.

Numerous tasks, including regression and predicting curvature fit, can benefit from neural networks. The artificial neural network will be used in this study as a forecasting model. A neuron is a basic building block of a neural network that utilizes a transfer function to produce an output. Next, each input is given through a weight, which serves as a connection between many layers of neurons and the transfer function to produce the desired output. The extensive structural architecture of the neural network system is depicted in Fig. 1. The network's main goal is to deliver a compressed revelation for the many erratic and patchy data, as opposed to the standard procedures like GD and LM, which devote minimal processing effort and demand prior understanding of statistical estimate among the variables^16^.

## Methodology

The stepwise methodology applied is as follows:

### Data compilation and study location and application of ANN

The data for relative humidity, irradiance, and ambient temperature^27^ has been gathered and compiled scrupulously to get the voltage output. Consequently, the input data has been splinted to test and train the data in order to process with the help of an LM-ANN approach. The gathered data for typically sunny and rapidly shifting climatic states of affairs, like partially cloudy, clear sunny day, and rainy day reveals an uncertain behaviour. The Pir Panjal region is that its weather varies and abruptly and unexpectedly. The circuitry was premeditated manually to collect the whole data within time intervals of milliseconds. The collected data elaborates that even a slight alteration in the ambiance^23^ can be observed at each moment. The entire range is blessed with enormous and gigantic mountains with a lot of solar power potential, but the abruptly altering weather conditions proffer big challenges to solar power installations. Therefore, such areas need precise model for extra-terrestrial forecasting so that the installed facility can be manoeuvred at utmost efficacy. The extra-terrestrial radiations and the long days can be calculated using relationships in the following equations. The whole setup includes a 100 Wp solar PV panel, a thermometer for observing cell temperature and ambient temperature^24^, and diffused and beam solar radiation measuring devices with their characteristics mentioned in the Table 2. The laboratory setup shown in Fig. 3 for a 12 V battery for the backup system was prepared to avoid any power supply disruption. The area under examination is profoundly subjective of the serene mediterranean environment right through the months of summer and consequently experience desiccated and shrivelled sweltering and sizzling within 3–4 months specifically; rain right through the year is usual. Hence, the western and north-eastern brutal winds manoeuvre the swot locality with soaring precipitation may result in hailstorms and snow as and when with chilly and wet days.

The preparation is intended to examine PV panels' broad spectrum and varying characteristics under climatic conditions. Besides, another facet is to mark the best chronological and metrological states for the generation of electrical energy by the PV panels. Designed for a minuscule time extension for forecasting the May, June, July, and August months, the data has been observed in a more detailed^25^ way. While, it has been observed that in the range of Pir Panjal, the 3–4 months when the vagueness lasts the most, as the thunderstorms and precipitation are pretty probable. Therefore, ambiguity is at its zenith all through this prediction. The lofty errors^8,30^ can be acknowledged as follows.

19 $${H}_{o=\frac{24}{\pi }}{I}_{sc}\left(1+0.033\mathit{cos}\frac{360N}{365}\right)X\left(\mathit{cos}\left(\varphi \right)\right)\mathit{cos}\left(\delta \right)\mathit{sin}\left({\omega }_{s}\right)\frac{2\pi {\omega }_{s}}{360}\mathit{sin}\left(\varphi \right)\mathit{sin}\left(\delta \right).$$

Here, Isc is the solar constant = 1367 W/m^2^, φ is latitude angle of area to be investigated, δ is the declination angle, ω_s_ is declination, and N is nth day of year. The declination angle can be calculated^9^ by:

20 $$\delta =23.45^\circ \mathit{sin}\left[\frac{360\left(N+284\right)}{365}\right],$$

21 $${\upomega }_{\mathrm{s}}={\mathrm{cos}}^{-1}\left[-\mathrm{tan}\left(\updelta \right)\mathrm{tan}\left(\mathrm{\varphi }\right)\right].$$

The use of the following relationship can attain the day length^10^:

22 $${\mathrm{S}}_{\mathrm{o}}=\frac{2}{15}{\upomega }_{\mathrm{s}}.$$

### Artificial neural network implementation and its performance

**Figure 2:**
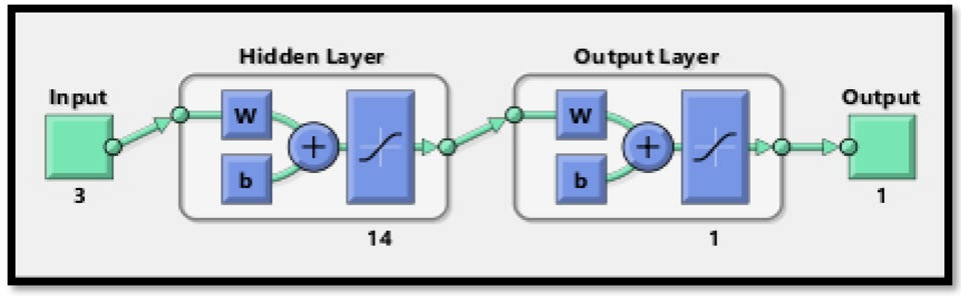
Training structure of an ANN model.

**Figure 3:**
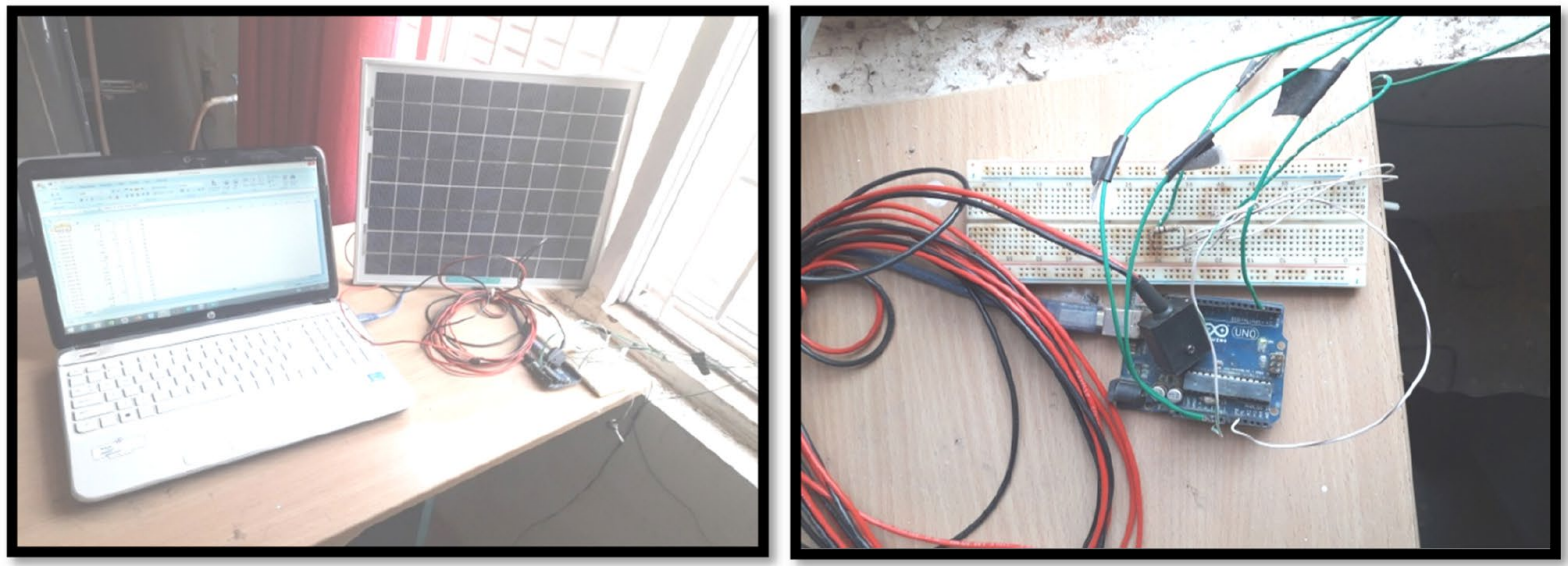
Laboratory arrangement for data recording for input variables.

The outline of neural network turns out to be quite essential while predicting global radiations. The neural networking model consists of three layering systems in which the input layer obtains the data collected. The lone output layer produces^12^ the information and may make use of one or more than one hidden layer essential for combining both layers via neurons. The output may be predictable by adequately training the input and output data in the ANN approach. GD and LM^15^ back propagation algorithms are used in a multiple layer’s feed-forward networking system. In this experimentation, the MATLAB 2016 version with three layered feed-forward networks operated with a sigmoid activation function and hidden layers, whereas the output layer uses the linear activation function. Three input variables, like ambient temperature, solar irradiance, and relative humidity, were applied; and output voltage was forecasted as an output. The predicted output by the proposed ANN model is shown in Table 3. The GD and LM-ANN algorithms^17,29^ have been applied to forecast the voltage at the output analogous to the collected data on *ms* basis. Figure 2 shows the neuron training structures. The LM-ANN algorithm features during the training process are listed in Table 1. The equipment and devices used are as follows in Table 2 (Fig. 3).

**Table 1:** LM-ANN algorithm and specifications.

Nature of the particular day	Rainy	P.Cloudy	Sunny
Uppermost epochs for training	1000	3000	2000
Goal of performance	0	0	0
Peak failures of validation	100	100	100
Smallest gradient of performance	1e_5	1e_7	1e_8
Preliminary mu	0.001	0.001	0.001
Descending mu factor	0.1	0.1	0.1
Ascending mu factor	10	10	10
Highest mu	1e10	1e10	10^10^
Displaying epochs	25	25	25
Generated command line output	False	False	False
GUI training	True	True	True
Greatest training time in seconds	Inf	Inf	Inf
Best validation performance	0.13367	0.012123	0.037915
Epochs adopted for validation	1000	676	582
Validation at epoch	998	576	422
No. of hidden layers used	2	2	2
No. of neurons used	14	14	14
No. r of output layers	1	1	1
Training function used	TLM	TLM	TLM
Adaption learning function	LGDM	LGDM	LGDM
Performance function	MSE	MSE	MSE

**Table 2:** Devices with their properties used in the PV system.

Device	Properties
Solar PV panel	100W
Pyranometer[GR]	[GR]:LP
Pyranometer[DR]	[DR]:LP
Thermometer	Comet system-T1110
Solar tracker	Lorentz-Etatrack Active 400 Model
PV cell	Monocrystalline silicon cell (100 W)
Softwares	MATLAB 2016/Teraterm/Anaconda
Arduino	Arduino Nano
Datalogger	System 16 channel MS5D model

### ANN algorithm


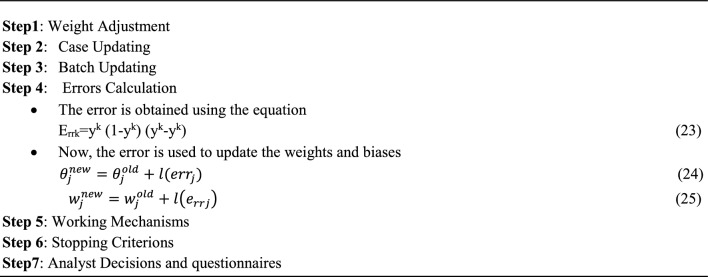


## Normalization, testing, and training

The neural network model engrosses the training of the input parameters by tuning the weights. This aspect intends to get training patterns competent enough to operate in optimum conditions. Furthermore, the model must be sufficiently capable of predicting the new information that does not emerge during the training, generating small errors while the testing set aspires to estimate the neural network performance^23,29^. This action is subjected to the input and output data sets normalized between the range of − 1 to + 1 preceding the training of the input parameters.

26 $${X}_{N}=0.8\frac{{X}_{R}-{X}_{max}}{{X}_{max}-{X}_{min}}+0.1.$$

Here, X_N_ is the normalized value, X_R_ is the normalization value, X_max_ is the maximum rate, and X_min_ is the lowest rate. After normalization progression, every input parameter is separated into two parts. The first part, called training, consists of 80%, and the second part, called the testing part, neural structure, is composed of 20% of the input variables.

## Selection of neurons in the hidden layers

It is hard to choose hidden layer neurons for the neural network. A single hidden layer is adequate for all the diverse functions. The neural network training is mostly initiated with an arbitrary^9^ weight assignment. Effectively, the number of neurons in hidden layers must be ascertained to reach the least error at the model's output. The choice depends on both Mean Square Error and 'R' values whenever the first one stays minimum and the later linear correlation coefficient^16^ remains high. To accomplish the neurons in the hidden layers, the neurons must be increased until they get united with Mean Square Error. Every single system possesses a solitary input layer and a lone output layer. The number of input parameters in the data processing mechanism is just equivalent to the number of neurons in the input layer. However, the number of outputs associated with every single input is also equivalent to the number of neurons in the output layer. The characteristic number in the data remains equivalent to the number of neurons in the input layer in some exceptional circumstances, for bias, there is only one input layer. Another condition which counts very much is that if the model is behaving like a classifier or a regressor very much depending upon the number of neurons in the output layer. The class labelling of the model also depends upon the condition that if it acts as a classifier, it must possess a lone neuron or might possess manifold neurons but on the contrary, if it behaves like a regressor then the output layer is supposed to have a single neuron.

**Figure 4:**
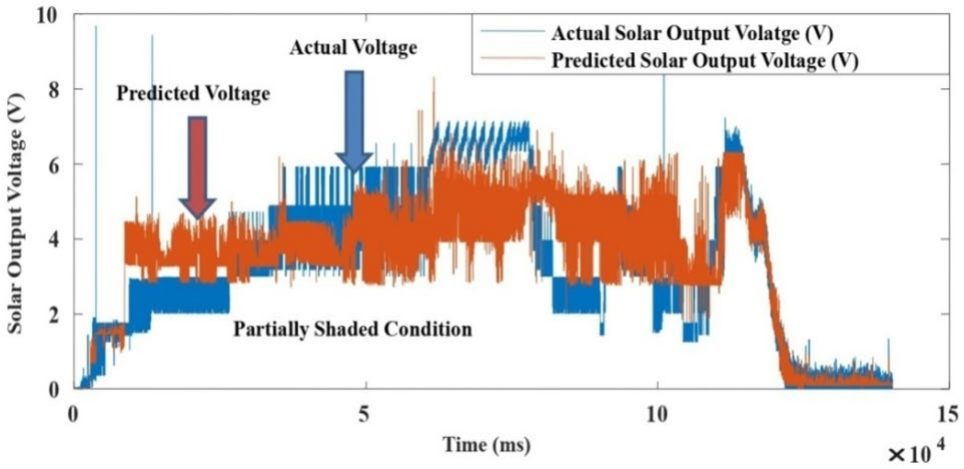
Comparison of actual and predicted solar output voltage with ANN for partially cloudy day.

**Figure 5:**
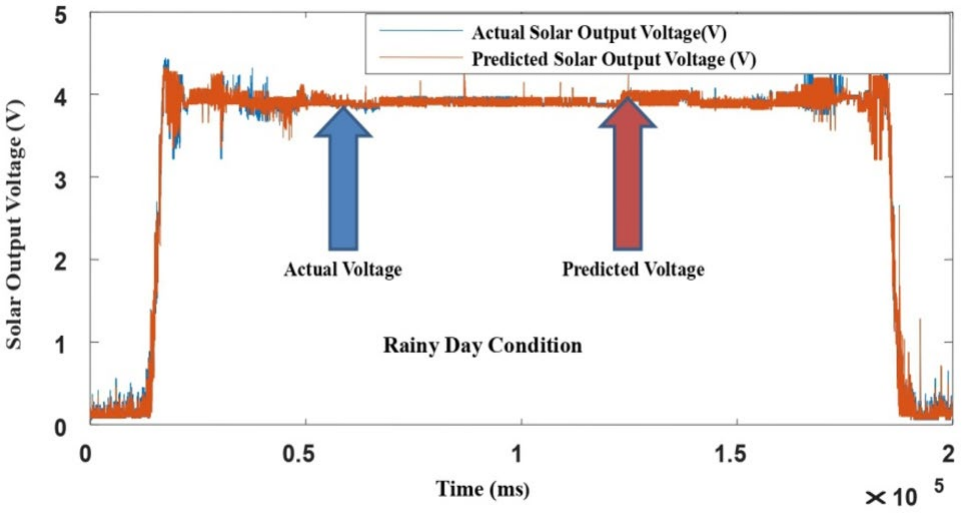
Comparison of actual and predicted solar output voltage with ANN for a rainy day.

**Figure 6:**
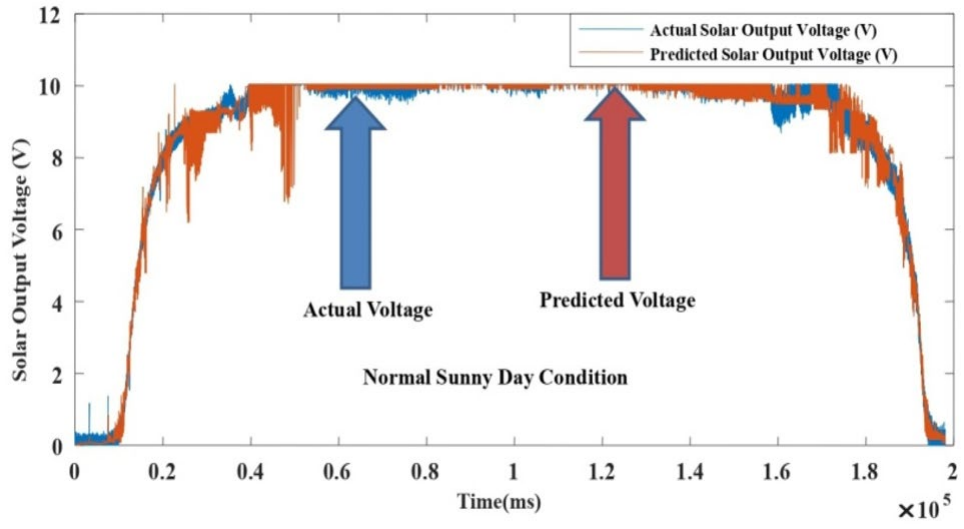
Comparison of actual and predicted solar output voltage with ANN for a sunny data.

The proposed LM-ANN approach creates R = 0.90, which means that if R > 0.90, the forecasted values with higher numbers are remarkably analogous to the collected data values. Thus, in this work, the LM-ANN approach^18^ has been preferred to conclude the selection of the total number of hidden layer neurons. The results show the least Mean Square Error^20,25^, and the maximum 'R-value was obtained at 14 neurons through both the testing and training stages, thereby confirming the exceptional Performance of the LM-ANN approach. Therefore, 14 neurons in the hidden layers are confirmed to be more fitting^5^ to predict the output voltage presented in Figs. 4, 5, and 6 for partially cloudy, rainy, and sunny days with the lowest MSE shown in Fig. 10a–c.

### Ethical approval

This article does not contain any studies with human participants or animals performed by any of the authors.

### Informed consent

Informed consent was obtained from all individual participants included in the study.

## Results and discussion

The LM-ANN model performances can be adjudged by the values of Mean Absolute Error, Root Mean Square Error and Co-efficient of correlation (R)^19,27^ by using the following relationships:

27 $$\mathrm{MAE}\hspace{0.17em}=\hspace{0.17em}\frac{1}{N}\sum_{i=1}^{N}\left|{X}_{i}-{Y}_{i}\right|,$$

28 $$\mathrm{RMSE}\hspace{0.17em}=\hspace{0.17em}\sqrt{\frac{1}{N}} {\sum_{i=1}^{N}\left({X}_{i}-{Y}_{i}\right)}^{2}.$$

Here, N is the entire data, Xi is the measured output voltage and Yi is the predicted output voltage by the LM-ANN approach. Figure 10a–c present the graphical demonstration of performance or error minimization plots with the lowest error. Figures 4, 5 and 6 compare the actual and predicted solar output voltage (V) waveform for partly cloudy, rainy, and sunny days.

## Error analysis

The outcome analysis shows the ease of the model. Nevertheless, the divergence at diverse locations can be evaluated by calculating the determination (C_2_D) and Mean Absolute Percentage Error using the relationships presented in the following equations.

29 $${\mathrm{C}}_{\mathrm{D}}^{2}=1-\left(\frac{{\sum }_{\mathrm{i}=1}^{\mathrm{N}}\left({\mathrm{X}}_{\mathrm{i}}-{\mathrm{Y}}_{\mathrm{i}}\right)}{\sum_{\mathrm{i}=1}^{\mathrm{N}}{\left({\mathrm{Y}}_{\mathrm{i}}\right)}^{2}}\right)\mathrm{and MAPE}= {\sum }_{\mathrm{i}=1}^{\mathrm{N}}\frac{\left|\frac{\left({\mathrm{X}}_{\mathrm{i}}-{\mathrm{Y}}_{\mathrm{i}}\right)}{{\mathrm{X}}_{\mathrm{i}}}\right|}{\mathrm{N}}\times 100.$$

Here, N is the data number, Xi is the measured output, and Yi is the forecasted output by the LM-ANN model.

**Figure 7:**
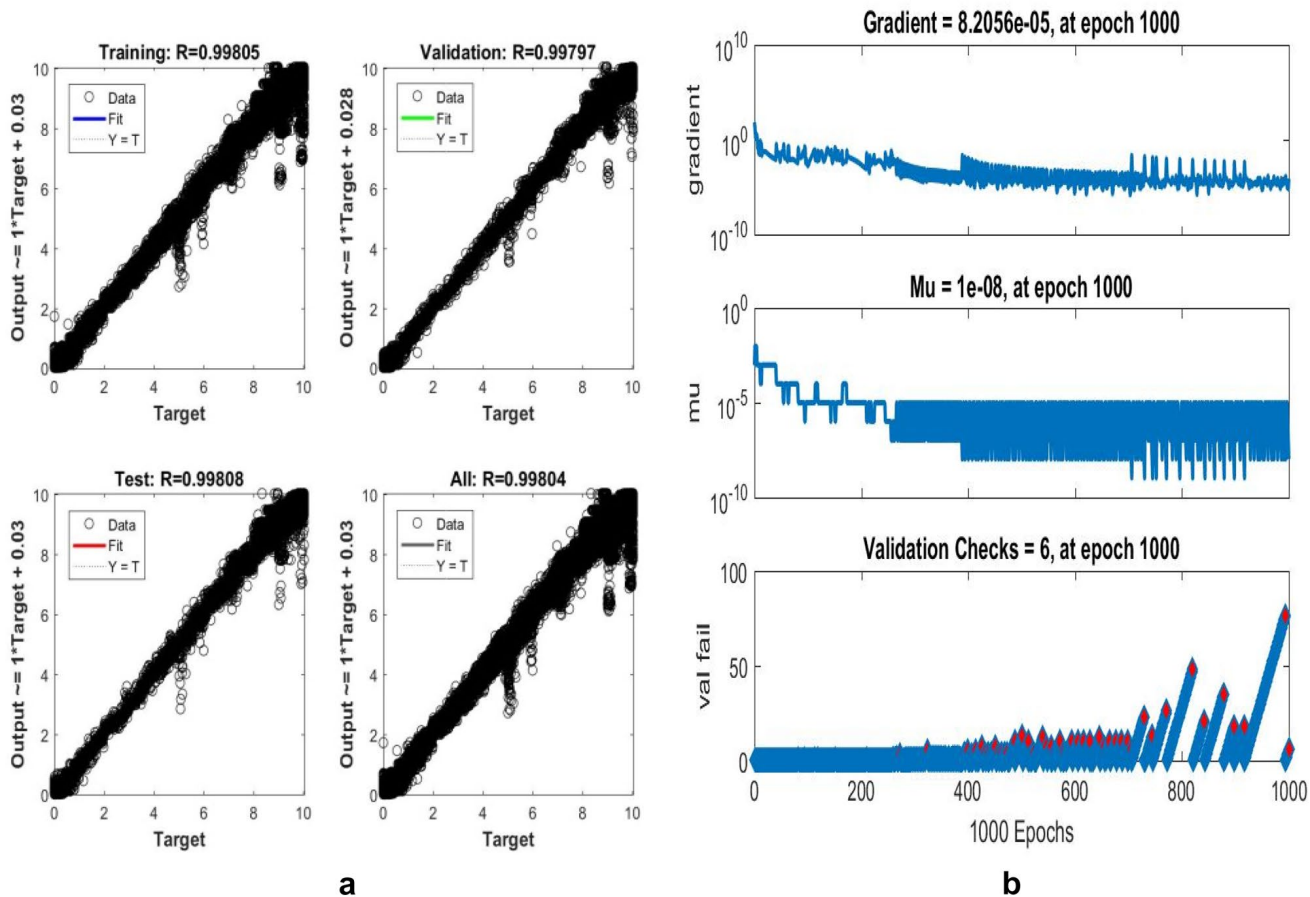
(**a**) regression plot (**b**) training plot for a sunny day.

**Figure 8:**
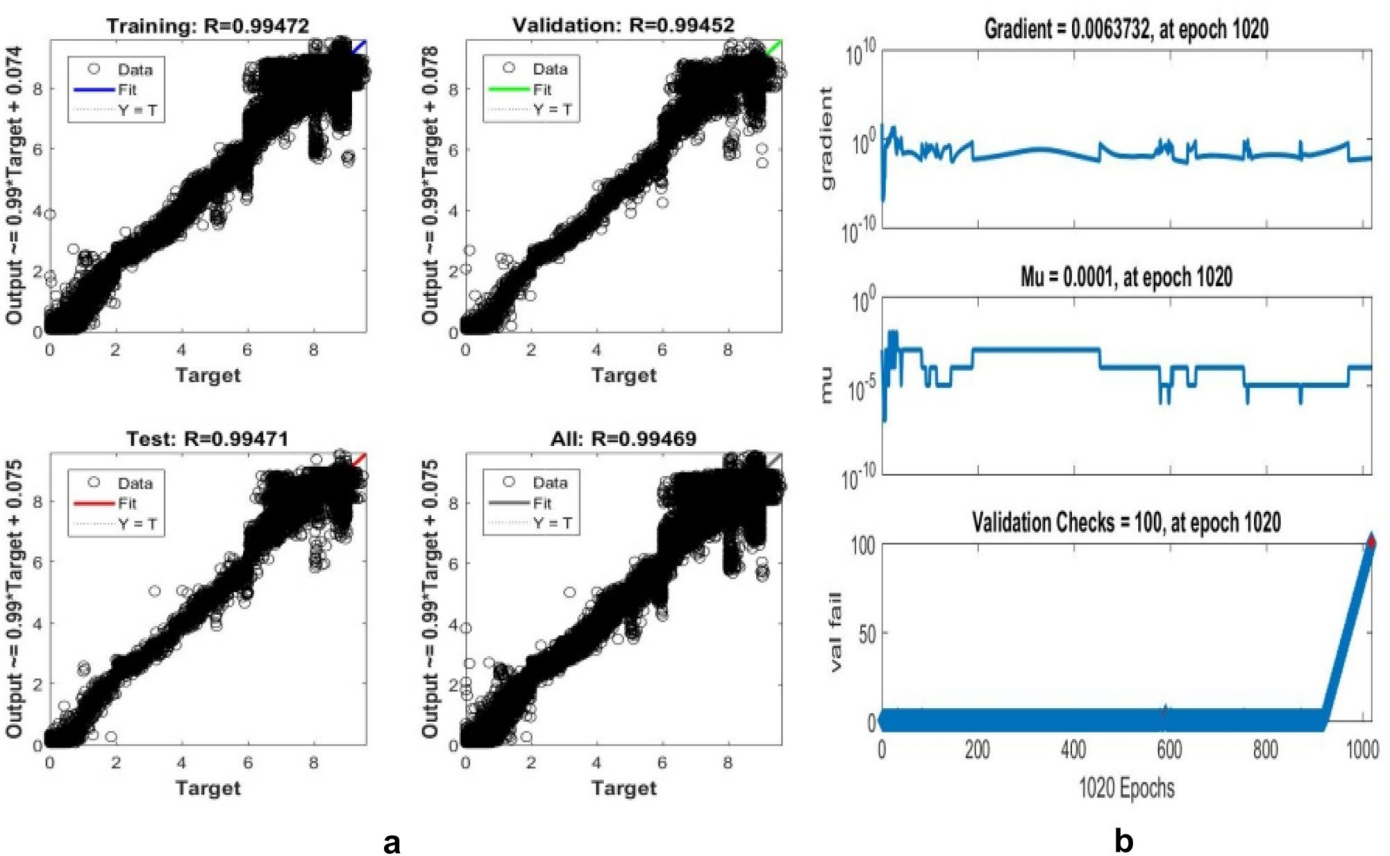
(**a**) Regression (**b**) training plot for a rainy day.

**Figure 9:**
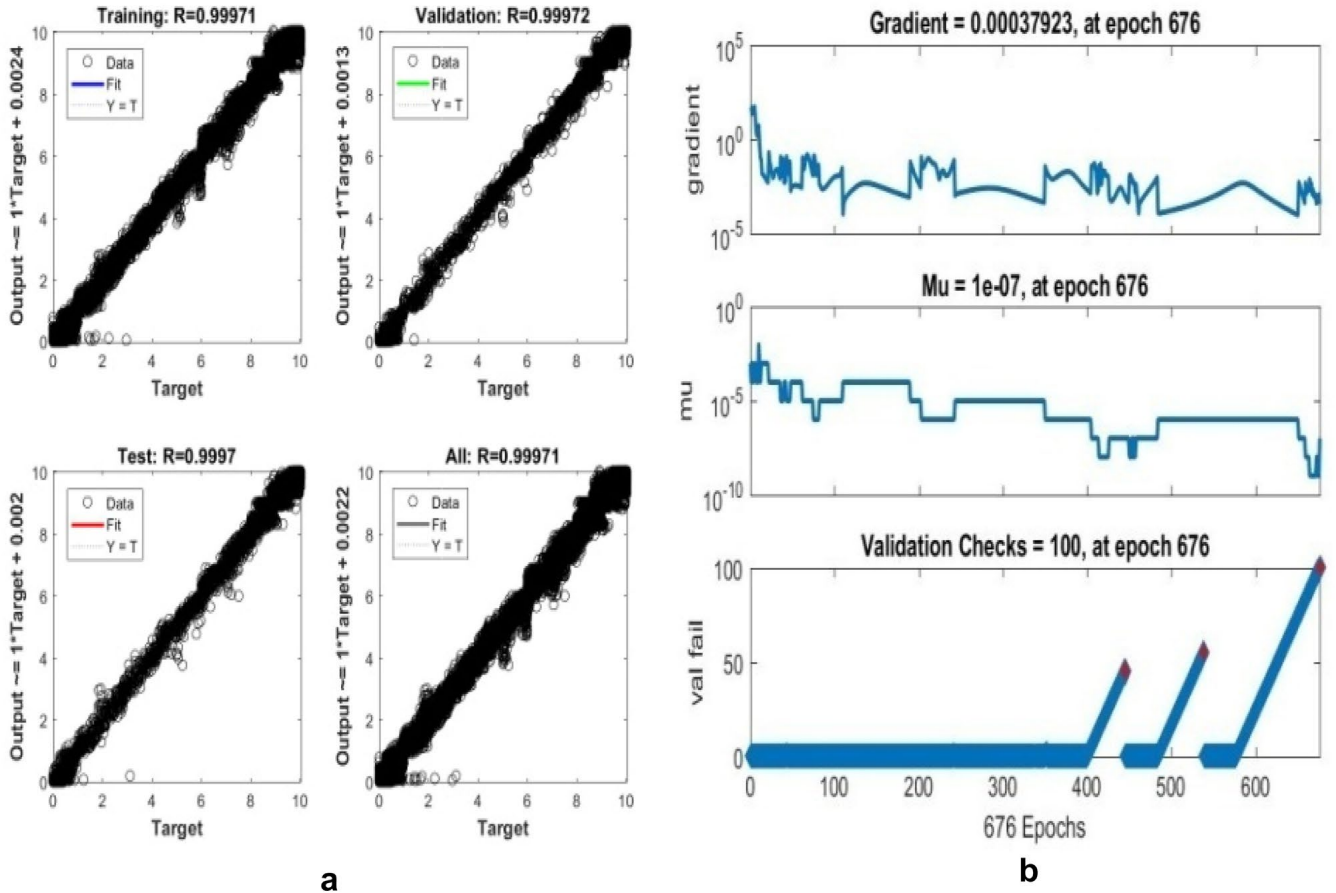
(**a**) Regression plot (**b**) training plot for diverse weather conditions.

Where mean absolute error provides the measure of resemblance between the measured and forecasted values, the Root Mean Square Error symbolizes the accuracy of the selected LM-ANN model. The Coefficient of correlation (R) value represents the correlation between the two values. If R = 1, then the linear connection between the two^6^ values can be acknowledged. Therefore, it can be developed that the model with the least values of Mean Absolute Error and Root Mean Square Error and a larger value of R advocates it as the best model to forecast the input parameters. The performances of the LM-ANN model for training and testing are presented in Table 3. The regression graphs both for the collected data and forecasted values by the LM-ANN^28,31,32^ for training and testing are shown in Figs. 7a,b, 8a,b, and 9a,b, correspondingly.

**Table 3:** Results obtained and comparison of two used models to train and test the data collected.

Algorithm used	ANN model (training)		ANN model (testing)			
MAE	RMSE	R	MAE		RMSE	R
Gradient descent ANN Model	Sunny day	5.7325	0.6755	8.2056	11.2127	0.5202
	Partially cloudy	6.2111	0.4324	0.0063	9.20232	0.4332
	Diverse weather	7.3260	0.4875	0.0003	8.3425	0.5763
Levenberg–Marquardt ANN Model	Sunny day	0.8327	0.8363	3.4979	4.5497	0.9223
	Partially cloudy	0.99472	0.99471	4.3213	4.4111	0.99808
	Diverse weather	0.99971	0.99970	3.3510	4.1231	0.99970

**Figure 10:**
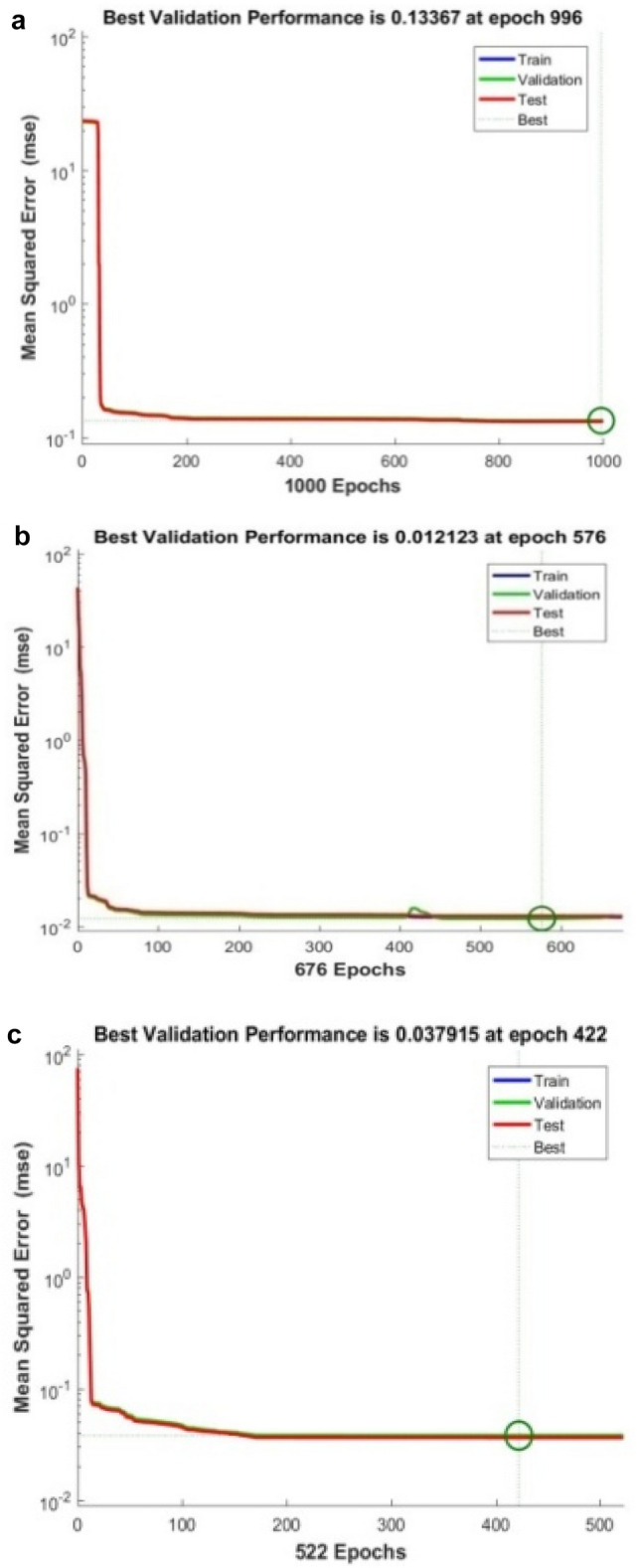
(**a**) Performance of rainy day (**b**) partially cloudy day (**c**) performance of a sunny day.

Also, R = 1 represents the perfect linear association between the collected and forecasted values, and R = 0.90 specifies an outstanding harmony^7^ between the two values. The proposed LM-ANN model presents 'R' values for both testing and training data as 0.9545 and 0.9272, thereby validating the claim of the LM-ANN approach.The numerical erroneousness divulges the least and maximum ${\mathrm{C}}_{\mathrm{D}}^{2}$ is 0.9808 and 0.9934, correspondingly. The Coefficient of determination represents the 97.9–98.9% correspondence between the forecasted and measured values by the proposed LM-ANN algorithm. The model performance for a sunny day, partly cloudy day, and rainy day are shown in Fig. 10a–c correspondingly. The MAPE value shows precision in the range of 0.11–4.24%. Therefore, from this mathematical error investigation, it is concluded that the precision of the LM-ANN approach^24^ obtained in this work has performed appropriately and absolutely to forecast the voltage at the output for the input parameters such as mean ambient temperature, solar irradiance, and relative humidity. The other characteristic of this approach is that it retorts very precisely and ideally on a shorter note, providing an excellent response with minimized error.

## Conclusion

In this work, the LM-ANN algorithm has been designed to forecast the output voltage in the region of Peer Panjal. The two ANN methodologies, GD and LM, were applied for testing and training purposes. The mean ambient temperature, relative humidity, and solar irradiance data were composed by the self-designed circuit in concurrence with Arduino to gather and survey the data at milliseconds. The hastily changing weathering data was used for training and testing the projected algorithm. The varied weather conditions like sunny days, rainy days, and partially cloudy day data were also used to test and train the LM-ANN technique. The reliability of the chosen technique was practically based on MAE, RMSE, and the Coefficient of maximum linear regression (R) value. The experimentation gives a much closer impression between the forecasted and the determined values. It also authenticates the exactness in the forecasting potential of the proposed technique. The numerical error study regarding the determination coefficient offers correspondence close to 98–99%. The MAPE value authenticates better precision too. One more vital aspect advocating the proposed approach is that it offers the promise and proficiency to congregate in a minute to tender, perfect revelation integrated with the slightest error.

## Data Availability

Data is available from the Neetan Sharma upon reasonable request.

## Abbreviations

ANN: Artificial neural network
ARF: Autoregressive fuzzy
ARIMA: Autoregressive integrated moving average
ARMA: Autoregressive moving average
DBN: Deep belief network
DRNN: Deep recurrent neural network
GD: Gradient descent
GHI: Global horizontal irradiance
HEV: Hybrid electric vehicle
LM: Levenberg Maquarndt
MA: Moving average
ML: Machine learning
MWNN: Mixed wavelet neural network
NWP: Numerical weather prediction
SAE: Stack auto encoder
